# Dissertation on the Diseases of the Gums

**Published:** 1845-12

**Authors:** E. Baker


THE AMERICAN

### JOURNAL OF DENTAL SCIENCE.

Vol. VI.] DECEMBER, 1845. [No. 2.

### ARTICLE I.

Messrs. Editors:—I am induced to make some preliminary remarks, because in the delivery of the following dissertation, there appeared to be a sentiment or an opinion, from a certain quarter at least, that it was objectionable, either in the spirit, manner, or matter, or perhaps all of them.

That it has many imperfections, I have not the least doubt; but to differ in scientific or practical opinions from any writer, however great and distinguished, can never be a mark of *disrespect;* nor when we consider the duties and expectations of man, can it be regarded as presumption to approach the sanctuary of knowledge and reconsider the ground and the validity of the most established doctrines, or of the most widely received opinions.

A distinguished writer has said, that all talents and all researches are but ministerial, to the improvement of true and practical knowledge and to the prevention, or the mitigation of human suffering by that auxiliar and uniting sympathy, by which the great system of the world in all its bearings and relations, amid every seeming irregularity and fancied deviation, is shown to be a system of benevolence.

It is a fact, that we owe the great bulk of our knowledge, not to those who have agreed, but to those who have differed; and those who have succeeded, by making all others think with them, have usually been those who began by *daring* to think for themselves; as it is said, he that leads a crowd must begin by separating himself some little distance from it. If the great Harvey, who discovered the circulation of the blood, had not differed from all the physicians of his day, all the physicians of the present day would not agree with him. Luther began by having his doubts, as to the assumed infallibility of the pope, and he finished by making himself the corner stone of the reformation.

Unity of opinion is not desirable, neither is it salutary, unless the truth be well established. It is important to remember also that assent or dissent is not the act of the will, but of the understanding. If we arrive at certain conclusions, and act conscientiously upon them, it would seem that no more should be required of us. We should certainly make use of *all* the means in our power to arrive at true conclusion, and let no interest warp us, no prejudice blind us, no party mislead us, nor any *fear* intimidate us. But there is a field of contention into which it is allowed, and it is good for man to enter, not with asperity, not with rudeness, not from a mere desire of distinction, nor from the rage and lust of gain; but from an honest endeavor to elicit what is true and what is useful, and from a desire not only allowable but laudable, to offer our common professional pretensions for honorable subsistence by honorable means.

In all sciences, in the medical, surgical, and dental science most particularly, the opinions of the greatest writers, and even of the greatest practitioners should be well weighed, and their mistakes, (if any are found or even suspected to exist,) should be pointed out for examination, with sincerity and with candor. To this there can be no objection, it is a duty which is owing to society, and to the usefulness of our profession. In particular, to those who devote themselves to dental surgery exclusively, it is indeed a duty paramount to every other consideration.

To living *professional* worth and reputation every tenderness is due; and while delicacy alone prevents my gratifying the desire which I should otherwise feel, of adverting to the merits (or possibly demerits) of many of my cotemporaries, I may be allowed to pay a passing tribute to departed excellence. Our departed Hayden opened many new sources of dental science, and who by his ability, by his judgment, and by his practice, enlarged the bounds of his art, and gave stability to his precepts; to which no one of us can have recourse without feeling personal obligation and unfeigned reverence. He was a man of long and extensive experience, of originality in thinking, of talents, and of genius; and under such impressions would I consider every memorial of his indefatigable mind, and every result of his curious and important investigations. He has, and he will forever have a bright and memorable name. He was the head and corner stone of our infant society, and felt great anxiety and responsibility for its success, while living, and his last aspirations probably were that those principles might be adopted and carried out, which would perpetuate it.

But it is not the name, nor the doctrine, nor the practice of Fouchard, of Bourdet, of Jourdain, of Hunter, of Fox, or of Hayden, which should guide us implicitly, but it is the truth and the result of actual facts, founded on knowledge and on repeated experiment, which can alone establish a course of practice, at once safe and efficacious. In this manner we shall best recommend and "worthily magnify" our profession, to which, we hope due honor *will* be paid, and when it is conscientiously exercised, kindness and gratitude are always due.

In the dissertation which I now respectfully offer to the consideration of our society, and of the public, I trust to have been actuated by these motives.

I have confirmed my observations and facts by my own experience and by my personal practice, and hope they will pass for what they are worth, and nothing more.

*Dissertation on the Diseases of the Gums.* Delivered before the American Society of Dental Surgeons, at their Sixth Annual Meeting, July, 1845. By E. BAKER, M. D., D. D. S.

It cannot have escaped the attention of the observing and intelligent, that the ætiology, or the doctrine of the cause of disease of the gums, is generally but little understood; and with those who have paid most attention to this subject, there appears to be a discrepancy of opinion as regards the cause of disease.

Affections of the gums, in former time, and indeed at the present day, are frequently called scorbutic. I shall attempt to show that this is almost entirely a misnomer, for any disease of the gums. Affections of the gums were generally called scorbutic till sometime in the 17th century, when the disease was called by other names, when some attempts were made to classify and distinguish the disease by other names.

Hunter, Fox, and indeed most of the modern writers have applied this term to diseases of the gums, generally, without pointing out any characteristic features, of a scorbutic nature.

In the first place we will give a short history of the disease called scurvy, and endeavor to show that the gums are never affected by it, unless as a secondary symptom, when the system is affected by a general taint.

The scurvy is a disease of a putrid nature, much more prevalent in cold climates than warm ones, and which chiefly affects sailors, and such as are shut up in besieged places, owing, as is supposed, to their being deprived of fresh provisions, and a due quantity of acescent food, assisted by the prevalence of cold and moisture, and by such other causes as depress the nervous energy, as indolence, confinement, want of exercise, neglect of cleanliness, much labor and fatigue, &c. These several debilitating causes, with the concurrence of a diet consisting principally of salted putrescent food, will be sure to produce this disease. The diagnostic symptoms are characterized by extreme debility; complexion pale and bloated, spongy gums, livid spots on the skin, breath offensive, adematous swellings in the legs, hæmorrhages, foul ulcers, fœtid urine, and extremely offensive stools.

All these are but symptoms of a general taint. Amongst these are the gums. So are they affected when the system is charged with mercury, and with the same propriety might a common disease of the gums be called a mercurial disease, when no mercury has been taken by the patient. Hence it would seem impossible that the gums of persons who are not in a situation to contract a scorbutic taint, should be scorbutically affected, as it will be recollected the "spongy gums" are the *result* only of the aforesaid taint.

Dr. Hayden, in his treatise on "conjoined suppuration," observes, that sometime in the 17th century, Fouchard wrote and published a work on the diseases of the mouth and teeth, and in which the disease in the gums, for the first time, was treated on distinctively, of which he, (Fouchard,) thus observes, "there is moreover another species of scurvy, of which I believe, as yet, no author has taken care to speak of, and without interesting any other part of the body, attacks the gums, the alveoli and the teeth.

Bourdet, it appears, called it "a suppuration of the gums."

This disease is described by Jourdain, who, perhaps, was the first to call it a "conjoined suppuration of the gums and alveoli."

Mr. Fox attempted to describe the disease under consideration, as one of the complaints to which the alveolar processes are liable. He says, "the most common disease to which the alveolar processes are subject, is a gradual absorption of their substance, whereby the teeth lose their support, become loose and drop out." This disease, he continues, "begins to show itself, between forty and fifty years of age, and from its frequent occurrence, without any evident cause, it would seem to be a consequence of having passed the middle of life! Thus he seems to have fallen into the common opinion, that persons advancing in age, *must* lose their teeth, in spite of all or any remedy—that it is as natural a consequence of advancing age, as death is of old age! But we think Mr. Fox is mistaken, as well as those who think with him, and would fain hope, that there is no such an "opprobrium" attached to *any* branch of our profession.

But to return to our subject. It will, perhaps, be unnecessary to follow those authors in their description of the symptoms and appearance of the gums, in this state of disease; but will just name some of the causes, mentioned by some of the French writers, either operating directly or indirectly, on this subject, viz. "a depraved or vitiated state of the circulating fluids in the parts affected, thereby rupturing the capillary vessels and causing numerous little ulcers, which were actually found to exist, according to Bourdet and Tennon, on the surface of the gums, next to the teeth, whence comes a purulent discharge from under the gums, so common in this disease. It was considered too, by Bourdet, as an erysipelatous affection.

Among the causes, remotely, are great exercise of the mind, melancholy, and bad diet, the sudden closing up or healing of issues, &c., &c. The sudden check of some prevailing cutaneous disease; the putrid miasma of low and humid places, of hospitals, and also the gaseous emanations from mines, &c.

Dr. Hayden seems to have followed, to some extent, the pathologic views of those writers on this subject, and observes, that the disease in question, though various in its character, is specific in its nature, peculiar in its operations and results, and in all cases, *primarily seated,* in the investing membranes or periosteum that surrounds the roots of the teeth, and lines the respective cavities of the alveoli, and he further observes that Jourdain, Bourdet, Ricci, and others, who have denominated it a conjoined suppuration of the alveoli and gums, have not been careful to explain *all* the different characters which it assumes, and have, therefore, treated it as one disease, and under one particular head.

Dr. Hayden divides this particular disease into three grades. "The first signs of this affection," he says, "are manifested by a bright circumscribed redness about the edge of the gums." But it is unnecessary to quote the doctor, in his description of the symptoms and characteristics of the disease, all of which may be found in his treatise on the subject of "conjoined suppuration."

The French writers, I believe, and Dr. Hayden we know, assert that the cause of the disease may arise from both internal and external causes. We shall contend for the *external* causes for this disease, and that external causes *alone* are sufficient, when neglected, to produce all the signs and symptoms and deplorable consequences contended for, in the "conjoined suppuration."

That which Dr. Hayden mentions as the first sign, that the disease is "*primarily seated* in the investing membranes or periosteum, viz. "a bright circumscribed redness about the edge of the gums, we consider nothing more than a *local* irritation of the gums in the beginning of its disease and that the disease does not reach the investing membranes and alveoli, until this primary affection of the gums has been long neglected, and if suffered to go on, will approach and embrace those parts, on which the teeth depend for support.

We think it cannot be proved, and is nothing more than a hypothesis, that the disease ever attacks the periosteum and alveoli *primarily*, or in its first stage. Those who give this pathologic view, give reasons which appear as not sufficient to produce this primary internal cause of disease, viz. such as "a vitiated state of circulating fluids, great exercise of mind, melancholy, bad diet, hæmorrhoides, the sudden healing up of setons, checking cutaneous diseases, miasmas, hospitals, gaseous emanations from mines, &c. And as regards women, premature and repeated suppression of milk, menstrual obstructions, and also such as have experienced a total suppression of the periodical ècoulemant, and cum multes alias, equally potent reasons.

It is in the recollection of all of us, that but a few years past, the advocates for internal decay of the bony structure of the teeth, in its commencement, having no connection with external causes, were common, and perhaps composed a majority of those who were capable of forming an opinion on the subject.

At this time, there is perhaps hardly a solitary individual who does not believe that external causes, together with malformations of the teeth, are altogether and alone sufficient to decay them, and that there is no evidence that decays begin from internal causes.

Whence this gradual change of opinion as regards the cause of decay in teeth? What but this, that after a long and careful examination of the nature and cause of decay, its beginning and progress, opinion has settled down into the belief that caries begins externally—perhaps without exception. Although it has been proven that the bony structure of the crowns of teeth have vessels ramifying through their various parts, yet we can discover no diseased state of the human system which renders it probable that a diseased action, sufficient to decay the internal bony structure, can be thrown upon those parts. Is there not some analogy in those cases? Can it be reasonable that unhealthy affections of the human system shall have greater power in imparting a diseased action *primarily* to the periosteum and alveoli, than *primarily* to do the same, to the internal bony structure of the crown of a tooth?

Having offered a few reasons endeavoring to prove that affections of the gums, periosteum and alveoli, proceed from external causes, we will examine into the propriety of calling a general disease of the gums, periosteum and alveoli, "a conjoined suppuration."

There are different kinds of suppuration, called pus, and which, according to its nature, is called good pus, scrofulous, serous and ichorous pus. The nature of ichorous pus is thin, aqueous and acrid. Of this kind is the pus discharged from the gums, especially in a protracted disease of them, and when it reaches the alveoli, acts on the bone as an absorbent. It is a contradiction of terms to say the alveoli (which are bone) suppurate. Therefore should not this compound disease, called by the French authors, "conjoined suppuration" of the gums, &c., be called a suppuration of the gums and the *absorption* of the alveoli? And now I put the question with confidence: Is not this ichorous pus which is engendered in diseased gums, sufficient, in all conscience, to produce gradually all those lamentable effects described by those writers who advocate the destruction of the appendages and supports of the teeth, from *other* causes?

If the French hypothesis be true, that the disease begins in the periosteum; it follows that there can be no direct treatment, for you cannot reach the cause. Now I have always found a direct treatment beneficial, except, perhaps, in the artificial disease, produced by mercury, or when there is a scorbutic taint in the system. I have no doubt the gums are affected more or less by the various diseases we are subject to; but not to that extent as is generally supposed.

It is remarked also by writers on the deep seated disease in the gums, that it attacks those persons who otherwise enjoy the best of health, and soundest teeth. This would seem to indicate that the liability to this disease is increased by fullness and gross habit of body. This perhaps is the case, but it is also a fact that no condition in life or habit of body, is exempt from this disease.

Having assumed that this disease, perhaps universally, commences externally with the gums and progresses to the internal parts, a diagnostical description may be expected, and a mode of treatment pointed out for its cure. I shall very briefly dwell on this part of the subject, which has been well described by Kœcker, in his chapter on the "absorption of the gums and the sockets of the teeth," and generally agree with him in his views of the subject, except, perhaps, I am unwilling to allow that the disease produces, so often and so much, constitutional derangement, as he describes.

The doctor says, "this malady has its beginning, generally, in an inflammation and suppuration of the gums, which gradually extend to the periosteum and the alveolar processes of the teeth; or it begins by an inflammation of those parts, which is afterwards communicated to the gums; it very rarely originates in the alveoli themselves."

To be sure, he says, the disease *may begin* in the periosteum and alveolus, though but very seldom. But the doctor does not attempt to show, like our French brethren, what induces this disease, *primarily*, in those parts, which we believe would be difficult. "The inflammation and suppuration are seldom violent, and the absorption seldom rapid; in most instances it is so slow in its progress, as to be scarcely perceptible; and suppuration destroys the gums in a very gradual manner, being attended by the absorption of the alveoli and their periosteum, until the teeth losing their support, become loose, and at successive intervals, drop out."

"The crowns, necks, and more especially the exposed parts of the roots, are frequently covered with a greenish glutinous substance, and with adhering tartar; the spaces between the teeth are filled up with tartar of a dark brown or greenish color, but sometimes they are of the usual appearance."

"In other instances I have seen them so clean as to deceive a superficial observer, but a close examination, has never failed to show some tartar adhering to the roots, and pressing on the alveolar processes, hidden under the edge of the gums, and in the spaces between the teeth."

As it respects symptoms, the doctor observes: "for a considerable time, even for many years, the symptoms accompanying this disease may entirely escape the attention of not only the patient, but of the surgical attendant not well acquainted with this disease. The matter which is discharged, is, in the first stage of the disease, very trifling, and constantly removed by saliva and mastication ; and the inconvenience and pain accompanying the malady, are so slight, and the progress of it so gradual and regular, that it may sometimes go on for ten years and upwards before it is observed."

After dwelling at some length on the constitutional derangements, nervous derangements, &c., he continues, "indeed, so great is the morbid influence of this malady, upon the general system, that after a perfect cure of the local disease has been effected, not only all those symptomatic affections subside, but the general health, for the first year or two, invariably improves in a most surprising manner, and the constitution recovers that natural strength and vigor, of which it has been deprived for perhaps ten or fifteen years.

Among some of the remote causes which he enumerates, are, "a scorbutic and scrofulous habit of the gums, use of mercury, irregularities in the position of the teeth, neglect of cleanliness, operations of different kinds, injudiciously performed," &c. By the above causes, "without which," he says, "he has never seen the disease ; a collection of tartar is deposited on particular parts of the teeth, and this becomes the immediate exciting cause of the disease, and so long as it is suffered to remain, prevents the success of such efforts as nature or medicine may make for the accomplishment of a cure."

I agree, in general, with his method of treatment, to his account of which, we all have access, and will add, that my own experience is, that it may take from one to six months, and perhaps more, to subdue this disease, according to its extent, by repeated operations, principally on the necks, and particularly on the fangs of the teeth. There is a deposit of tartar on the fangs of the teeth, which penetrates as low as the same is separated from the gum and periosteum, and until this is entirely removed a perfect cure cannot be expected. This, in my opinion, is the exciting cause of the disease; for on removing *all* this matter from the fangs of the teeth, the cure follows rapidly. I consider that this state of the teeth and investing parts, requires, by far more time and patience, both of the patient and operator, than is generally supposed; great experience and tact, in the operator, supplied with proper instruments for the purpose. All astringents, powders, brushes, and appliances in the world, will not remove the *cause* of disease; this is only to be effected by *instruments*, and the other means to produce cleanliness and to assist in giving healthy action to the parts, are but of secondary importance, but also highly important.

As Dr. Kœcker justly observes, "considerable experience and skill are required, to distinguish at *once* between those teeth which are capable of preservation, and such as ought to be extracted."

I will observe, that it is much easier to treat successfully the front teeth than those farther back, when apparently affected to the same degree, arising from the facility with which they can be operated on. The disease in the incisors can be cured, or kept down, when quite loose, which is not the case with the molars.

I consider it unnecessary in an essay of this kind to be prolix. Happy shall I be if I can be instrumental in turning the attention of the fraternity to this subject, a subject which is acknowledged to be but "imperfectly understood," and by far the most difficult of treatment. We are greatly indebted to *all* writers on this subject; not a little to the French authors, who have most learnedly and prolixly written on this subject, and whose disquisitions, in many respects, remind us forcibly of the story of the child with the golden tooth, followed up by a number of ingenious theories, by different writers, endeavoring to account for the same.

It is an old maxim that those disorders which have no cure, are the favorite ones for quacks to be engaged in, and that those sciences, in which there is any degree of uncertainty, is sure to employ the most quills.

So it appears that those sciences that are capable of being demonstrated, or that are reduced to the severity of calculation, are never voluminous; for clearness is intimately connected with conciseness, but precisely in proportion as certainty vanishes, verbosity abounds. But when we consider that our

French brethren were perhaps the first to write on this subject, as well as others relating to the teeth, much might be said in palliation of inaccuracies, and it would be strange if they were as well acquainted with the doctrine of the causes of dental diseases as we *ought* to be at this period of time ; and I have no doubt they may come in and partake of the following sentiment, viz. the discovery of truth in art or science, should be the polar star, to which our attention should always be directed, and those who contribute to the establishing of it in the remotest degree, deserve the thanks of mankind.

